# Problematic internet use and psychiatric co-morbidity in a population of Japanese adult psychiatric patients

**DOI:** 10.1186/s12888-018-1588-z

**Published:** 2018-01-17

**Authors:** Hille T. de Vries, Takashi Nakamae, Kenji Fukui, Damiaan Denys, Jin Narumoto

**Affiliations:** 1 Academic Medical Center, University of Amsterdam, Meibergdreef 9, 1105 AZ Amsterdam, the Netherlands; 2 Department of Psychiatry, Graduate School of Medical Science, Kyoto Prefectural University of Medicine, 465 Kajii-cho, Kawaramachi-Hirokoji, Kamigyo-ku, Kyoto, 602-8566 Japan; 3 Health Care Center, Kyoto Prefectural University of Medicine, 465 Kajii-cho, Kawaramachi-Hirokoji, Kamigyo-ku, Kyoto, 602-8566 Japan; 4 Department of Neuropsychiatry, Academic Medical Center, University of Amsterdam, Meibergdreef 9, 1105 AZ Amsterdam, the Netherlands; 5 The Netherlands Institute for Neuroscience, an institute of the Royal Netherlands Academy of Arts and Sciences, Meibergdreef 47, 1105 BA Amsterdam, the Netherlands

**Keywords:** Internet addiction, Problematic internet use, Prevalence, Japan

## Abstract

**Background:**

Many studies reported the high prevalence of problematic internet use (PIU) among adolescents (13–50%), and PIU was associated with various psychiatric symptoms. In contrast, only a few studies investigated the prevalence among the adult population (6%). This study aimed to investigate the prevalence of PIU and psychiatric co-morbidity among adult psychiatric patients.

**Methods:**

Three hundred thirty-three adult psychiatric patients were recruited over a 3-month period. Two hundred thirty-one of them completed the survey (response rate: 69.4%, 231/333; Male/Female/Transgender: 90/139/2; mean age = 42.2). We divided participants into “normal internet users” and “problematic internet users” using a combination of Young’s Internet Addiction Test (IAT) and the Compulsive Internet Use Scale (CIUS). Demographic data and comorbid psychiatric symptoms were compared between the two groups using self-rating scales measuring insomnia (Athens Insomnia Scale, AIS), depression (Beck Depression Inventory, BDI), anxiety (State-trait Anxiety Inventory, STAI), attention deficit hyperactivity disorder (ADHD) (Adult ADHD Self-report Scale, ASRS), autism (Autism Spectrum Quotient, AQ), obsessive-compulsive disorder (OCD) (Obsessive-Compulsive Inventory, OCI), social anxiety disorder (SAD) (Liebowitz Social Anxiety Scale, LSAS), alcohol abuse, and impulsivity (Barratt Impulsive Scale, BIS).

**Results:**

Among 231 respondents, 58 (25.1%) were defined as problematic internet users, as they scored high on the IAT (40 or more) or CIUS (21 or more). The age of problematic internet users was significantly lower than that of normal internet users (*p* < 0.001, Mann–Whitney U test). The problematic internet users scored significantly higher on scales measuring sleep problems (AIS, 8.8 for problematic internet users vs 6.3 for normal internet users, *p* < 0.001), depression (BDI, 27.4 vs 18.3, *p* < 0.001), trait anxiety (STAI, 61.8 vs 53.9, p < 0.001), ADHD (ASRS, part A 3.1 vs 1.8 and part B 3.5 vs 1.8, *p* < 0.001), autism (AQ, 25.9 vs 21.6, *p* < 0.001), OCD (OCI, 63.2 vs 36.3, *p* < 0.001), SAD (LSAS, 71.4 vs 54.0, *p* < 0.001), and impulsivity (BIS, 67.4 vs 63.5, *p* = 0.004).

**Conclusions:**

The prevalence of PIU among adult psychiatric patients is relatively high. As previous studies reported in the general population, lower age and psychiatric comorbidity were associated with PIU among adult psychiatric patients. More research is needed to determine any causal relations between PIU and psychopathological illnesses.

**Electronic supplementary material:**

The online version of this article (10.1186/s12888-018-1588-z) contains supplementary material, which is available to authorized users.

## Background

With internet access and use pervading society worldwide, concerns have been raised about the potential damage of excessive internet use [1]. ‘Internet Addiction (Disorder)’, ‘Problematic Internet Use’, ‘Pathological Internet Use’, ‘Internet Addictive Behaviour’ and other terms have been used to describe the combination of addiction-like symptoms and social problems seen in individuals spending huge amounts of time using the internet [2, 3]. However, this proposed disorder is still under discussion concerning its definition, validity as a construct, proposed diagnostic criteria, preferred measuring instruments, and treatment [4, 5]. Proponents of the disorder have made considerable efforts to see it included in the American Psychiatric Association (APA)‘s Diagnostic and Statistical Manual of Mental Disorders (DSM), resulting in the inclusion of Internet Gaming Disorder in section III under the heading “Conditions for Further Study” [6].

Previous epidemiological studies used some measurements including the Young Diagnostic Questionnaire (YDQ), the Internet Addiction Test (IAT), and the Compulsive Internet Use Scale (CIUS) to assess excessive internet use. High prevalence of internet addiction among adolescents and students has been reported by many authors. Cheng et al. [7] conducted a meta-analysis including 89.281 participants from 31 nations and estimated prevalence of internet addiction defined as a YDQ score from 5 to 8 or an IAT score from 70 to 100 to be 6.0% among adolescents and youth. Tsitsika et al. [8] reported that prevalence of dysfunctional internet behavior defined as an IAT score from 40 to 100 among 14–17-year old students to be 13.9% in European countries (*N* = 13.284) while Mak et al. [9] reported its prevalence to range from 13.7 to 50.9% among 12–18-year-old students in Asian countries (*N* = 5.366).

In contrast, only a few studies investigated the prevalence among the adult population. Bakken et al. [10] reported the prevalence of internet addiction (YDQ score 5–8) and at-risk internet users (YDQ score 3–4) to be 1.0 and 5.2% respectively in 3.399 Norwegian subjects. Zadra et al. [11] reported that 685 out of 15.023 subjects (4.5%) showed 21 points and more on the CIUS. Thus, the problem of excessive internet use in adults should not be underestimated, even though its prevalence may be lower in adults than among students.

A number of studies have identified the correlation between problematic internet use (PIU) and psychiatric disorders [12–16], including a systematic review by Carli et al. [3] and most notably a meta-analysis by Ho et al. [2]. PIU was shown to be associated with alcohol abuse, attention deficit hyperactivity disorder (ADHD), depression, and anxiety. The correlation with depression has also been found in longitudinal studies [17–19], with individuals overly exposed to the internet having a higher risk of developing depression. Furthermore, PIU has also been found to correlate with sleep problems and these also have their effects on patients’ well-being [20–23]. Therefore, we deemed it necessary to assess the correlation between PIU and comorbid psychiatric symptoms among patients with psychiatric disorders as their psychiatric symptoms could affect PIU or vice versa.

As far as we know, however, there have been no studies as of yet investigating the prevalence and comorbidity of PIU in a psychiatric population. Psychiatric symptoms might either induce PIU in patients with psychiatric illnesses, or PIU might induce or aggravate psychiatric symptoms. Therefore, this study aims to investigate the prevalence of PIU among adult psychiatric patients, and to reveal the degree of co-morbidity between PIU and psychiatric symptoms.

We hypothesize that the prevalence of PIU among psychiatric patients to be higher than among the general population. PIU might be associated with sleep problems, depression, anxiety, ADHD, autism, obsessive-compulsive disorder (OCD), social anxiety disorder (SAD), alcohol abuse, and impulsivity as previous studies reported in adolescents and students.

## Methods

### Study design and population

A cross-sectional study was performed using online questionnaires. Participants were recruited through their treating physicians at the outpatient clinic of psychiatry, University Hospital, Kyoto Prefectural University of Medicine from January 2016 to April 2016, for which Kyoto Prefectural University of Medicine Research Ethics Committee approved all procedures (ERB-C-485). After obtaining both oral and written consent, participants were asked to fill in an electronic questionnaire either at home or on tablets provided by the researchers.

### In- and exclusion criteria

Participants were included if they had been diagnosed with a psychiatric diagnosis from the tenth revision of the International Classification of Diseases and Related Health Problems (ICD-10) [24] categories F1 (Mental and behavioural disorders due to psychoactive substance use), F2 (Schizophrenia, schizotypal and delusional disorders), F3 (Mood [affective] disorders), F4 (Neurotic, stress-related and somatoform disorders), or F5 (Behavioural syndromes associated with physiological disturbances and physical factors).

Participants were excluded if they were below 20 years of age, did not have access to the internet, or were in such a clinically bad shape that their treating physician preferred not to ask them to fill out the questionnaire.

In order to prevent and assess possible selection bias, whether or not the patient met the inclusion criterion for diagnosis or had to be excluded due to age was judged beforehand by the researchers by accessing the medical files for each patient scheduled for an appointment at the psychiatry outpatient department. If any of the treating physicians were unable to recruit a patient due to the patient’s clinical condition being too severe (i.e. the patient was not able to fill out the questionnaire), the patient not agreeing to participate in the research, or not turning up for their appointment, physicians were asked to note this on a form provided to them by the researchers.

### Measurements

To assess internet addiction, we administered both IAT developed by Young [25] and the CIUS developed by Meerkerk et al. [26], both translated into and validated in Japanese [27]. There seem to be different versions of the IAT in use. The validated Japanese version used in this research has 20 questions that can be answered on a Likert-scale of 1 to 5, with proposed cut-off points of 40 (problematic internet use), as also used in the large studies by Tsitsika et al. [8] and Mak et al. [9]. The cut-off for PIU on the CIUS was defined as 21 points, as suggested by Guertler et al. [28]. To measure sleep problems, we administered the validated Japanese version of the Athens Insomnia Scale [29, 30]. To measure the severity of other symptoms of mental disorders, we administered the validated Japanese versions of the Beck Depression Inventory II (BDI-II) [31, 32], the State-Trait Anxiety Inventory (STAI) [33, 34], the Adult ADHD Self-report Scale (ASRS) [35], the Autism Spectrum Quotient (AQ) [36, 37], the Obsessive-Compulsive Inventory (OCI) [38, 39], the Liebowitz Social Anxiety Scale (LSAS) [40, 41], the Alcohol Use Disorders Identification Test (AUDIT) [42, 43], the Barratt Impulsive Scale (BIS) [44, 45], and questions regarding the amount and nature of internet use and the patients’ background (Appendix).

The physicians diagnosed their patients based on ICD-10 criteria, and then assessed the severity using the Clinical Global Impression Severity (CGI-S) [46].

Data was only sent when participants completely finished the questionnaire. Any participants who might have completed only part of the questionnaire were thus counted as non-respondent, as we did not receive any of their data.

### Statistical analysis

Continuous variables were shown as mean (± standard deviation) or median (interquartile range), and categorical variables were presented as numbers and percentages. To study statistically significant differences between problematic internet users and normal internet users, we divided our sample in two groups. Respondents who scored 40 or higher on the IAT [8, 9] or 21 or higher on the CIUS [28] were defined as problematic internet users, while those who scored below those values on both were defined as normal internet users. Categorical variables were compared between two groups using chi-square test while Fisher’s exact test was performed in tables larger than 2 × 2 using R version 3.3.1 (http://www.r-project.org/). As results apparently didn’t follow a Gaussian distribution, differences in ordinal and interval variables between the two groups were assessed using the Mann–Whitney U test. The software used was SPSS for Windows version 23. Bonferroni correction was applied for multiple comparisons.

## Results

### Demographics and internet use

**Fig. 1:**
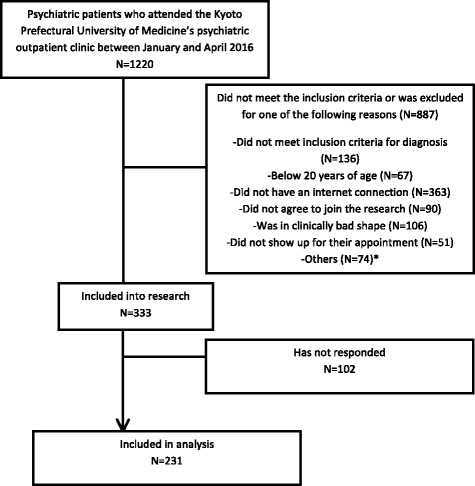
Flowchart of the inclusion of patients

As shown in Figs. 1, 1.220 patients visited the hospital during the 3 months of our recruitment period and 333 of them were included in this study. Two hundred thirty-one patients completed the survey, making the response rate at 69.4% (231/333). Fifty patients (21.6%, 50/231) scored above the 40 points thresholds on the IAT. Furthermore, 43 patients (18.6%, 43/231) scored 21 or higher on the CIUS. This brought the total number of patients we defined as “problematic internet users”, those who scored either 40 or higher on the IAT or 21 or higher on the CIUS, to 58 (25.1%, 58/231).

The proportion of problematic internet users is remarkably higher in the younger age groups and unemployed group (Table 1). Sex, education level, living alone or with others, and marital status do not seem to significantly affect internet addiction scores. Furthermore, PIU did not seem to differ greatly between the ICD-10 categories of the respondent’s main diagnosis (as reported by their treating physicians). CGI-S scores of patients with PIU assessed by their treating physicians were significantly higher than those of normal internet users.

**Table 1:** Demographics of the respondents

		Normal internet users (*N* = 173)	Problematic internet users (*N* = 58)	*p*
Sex	Male	69 (39.9%)	21 (36.2%)	0.10^a^
	Female	104 (60.1%)	35 (60.3%)	
	Transgender	0	2 (3.4)	
Age	Mean, years (standard deviation)	43.6 (12.7)	35.9 (11.9)	<0.001^b^**
	20–29	26 (15.0%)	23 (39.7%)	
	30–39	37 (21.4%)	14 (24.1%)	
	40–49	60 (34.7%)	12 (20.7%)	
	50–59	29 (16.8%)	6 (10.3%)	
	60–69	16 (9.2%)	3 (5.2%)	
	70–79	5 (2.9%)	0	
Education (highest level)	Junior high school	8 (4.6%)	5 (8.6%)	0.59^a^
	High school	57 (32.9%)	23 (39.7%)	
	Junior college	25 (14.5%)	6 (10.3%)	
	University	76 (43.9%)	22 (37.9%)	
	Graduate school	7 (4.0%)	2 (3.4%)	
Employment status	Employed/homemaker/studying	113 (65.3%)	29 (50.0%)	0.04^c^*
	Unemployed	60 (34.7%)	29 (50.0%)	
Living situation	With others	138 (79.8%)	47 (81.0%)	0.84^c^
	Alone	35 (20.2%)	11 (19.0%)	
Marital status	Married/living together	67 (38.7%)	17 (29.3%)	0.20^c^
	Unmarried/divorced/separated/widowed	106 (61.3%)	41 (70.7%)	
Diagnosis	ICD-10 F1	4 (2.3%)	1 (1.7%)	0.79^c^
	ICD-10 F2	20 (11.6%)	6 (10.3%)	0.80^c^
	ICD-10 F3	56 (32.4%)	17 (29.3%)	0.66^c^
	ICD-10 F4	94 (54.3%)	37 (63.8%)	0.21^c^
	ICD-10 F5	13 (7.5%)	3 (5.2%)	0.54^c^
Clinical Global Impression-Severity	Median (interquartile range)	3 (3–4)	4 (3–4)	0.009^b^*

Number of persons in each sex, age, education, employment, living situation, marital status, and diagnosis category

^a^Fisher’s exact test

^b^Mann-Whitney U test

^c^Chi-square test

*: *p* < 0.05, **: *p* < 0.001

### Psychiatry scale scores

Problematic internet users scored higher on all scales measuring co-morbid psychiatry showing a significantly uneven distribution of scores between the problematic and normal internet users, with the exception of the state subscale of STAI and AUDIT (Table 2).

**Table 2:** Average scores (and standard deviation) for the different scales measuring psychopathological co-morbidity of normal internet users compared with problematic internet users

	Normal internet users (*N* = 173)	Problematic internet users (*N* = 58)	*p* (Mann–Whitney U test)
Athens Insomnia Scale	6.3 (4.5)	8.8 (4.8)	<0.001*
Beck Depression Inventory	18.3 (12.7)	27.4 (11.9)	<0.001*
State-trait Anxiety Inventory (State)	49.3 (12.1)	54.0 (12.0)	0.013
State-trait Anxiety Inventory (Trait)	53.9 (11.0)	61.8 (8.5)	<0.001*
Adult ADHD Self-report Scale part A	1.8 (1.5)	3.1 (1.6)	<0.001*
Adult ADHD Self-report Scale part B	1.8 (2.3)	3.5 (2.5)	<0.001*
Autism Spectrum Quotient	21.6 (7.1)	25.9 (7.1)	<0.001*
Obsessive-compulsive Inventory	36.3 (31.7)	63.2 (34.1)	<0.001*
Liebowitz Social Anxiety Scale	54.0 (33.9)	71.4 (32.2)	<0.001*
Alcohol Use Disorders Identification Test	3.2 (4.4)	4.3 (5.5)	0.357
Barratt Impulsiveness Scale	63.5 (8.2)	67.4 (8.2)	0.004*

*P*-values represent the significance of uneven distribution between normal and problematic internet users on each of the scale scores, according to the Mann–Whitney U test

*Significant after Bonferroni correction

### Sleep habits

Some of the questions regarding to internet use (Appendix) revolved around the amount of internet use before sleep. The question “After you’ve gone to bed but before you sleep, how much on average per day do you use the smartphone or tablet?” (median < 10 min vs 30 min-1 h, Mann–Whitney U test, *p* < 0.001) and “When you wake up during your sleep, how much do you use your smartphone or tabled in bed?” (median “I don’t use it at all” vs “I hardly use it”, Fisher’s exact test, p < 0.001) got significantly higher answers from problematic internet users than from normal internet users (Additional file 1: Tables S1 and S2).

### Internet activities

While problematic internet users do not use the internet for work significantly more than normal internet users, they do use it more for private purposes (median 1 h–3 h vs >3 h, Mann–Whitney U test, p < 0.001) (Table 3). Several private activities on the internet seemed more closely associated with PIU (Additional file 1: Table S3). Problematic internet users seem to spend more private time on the internet for the purposes of communicating, looking up information, reading blogs/forums or making blog/forum posts, surfing social networking sites, and watching videos or listening to music.

**Table 3:** Reported time spent for work and private related activities on the internet among problematic internet users compared with normal internet users

Activities	Normal internet users, n (%) (*N* = 173)						Problematic internet users, n (%) (*N* = 58)						*p* (Mann–Whitney U test)
	None	<30 m	30 m-1 h	1 h–3 h	3 h–5 h	>5 h	None	<30 m	30 m-1 h	1 h–3 h	3 h–5 h	>5 h	
Work	51(29.5)	40(23.1)	31(17.9)	26(15.0)	11(6.4)	14(8.1)	25(43.1)	10(17.2)	6(10.3)	6(10.3)	4(6.9)	7(12.1)	0.300
Private	10(5.8)	23(13.3)	27(15.6)	69(39.9)	26(15.0)	18(10.4)	6(10.3)	1(1.7)	3(5.2)	12(20.7)	7(12.1)	29(50.0)	<0.001*

*: *p* < 0.001

### Other results

We also asked about the different devices participants used to access the internet (Appendix). Of these devices, only smartphone use was reported significantly more often by problematic internet users than normal internet users in all samples (median “I often use it” vs “I use it really often”, Fisher’s exact test, *p* < 0.001) (Additional file 1: Table S4). When the data was analyzed by age, only the twenties showed the significant difference in smartphone use frequency between the two groups (Table 4). However, this finding was not significant after Bonferroni correction.

**Table 4:** Comparison of smartphone use frequency between normal internet users and problematic internet users

Age	Smartphone use frequency	Normal internet users, n (%) (*N* = 173)	Problematic internet users, n (%) (*N* = 58)	*p* (Fisher’s exact test)
Total (*N* = 231)	I don’t use it at all	48 (27.7)	13 (22.4)	<0.001**
	I hardly use it	8 (4.6)	1 (1.7)	
	I sometimes use it	21 (12.1)	0	
	I often use it	40 (23.1)	2 (3.4)	
	I use it really often	56 (32.4)	42 (72.4)	
20–29 (*N* = 49)	I don’t use it at all	2 (7.7)	2 (8.7)	0.016*
	I hardly use it	1 (3.8)	0	
	I sometimes use it	2 (7.7)	0	
	I often use it	8 (30.8)	1 (4.3)	
	I use it really often	13 (50.0)	20 (87.0)	
30–39 (*N* = 51)	I don’t use it at all	5 (13.5)	3 (21.4)	0.12
	I hardly use it	3 (8.1)	0	
	I sometimes use it	2 (5.4)	0	
	I often use it	9 (24.3)	0	
	I use it really often	18 (48.6)	11 (78.6)	
40–49 (*N* = 72)	I don’t use it at all	20 (33.3)	4 (33.3)	0.14
	I hardly use it	3 (5.0)	0	
	I sometimes use it	11 (18.3)	0	
	I often use it	12 (20.0)	1 (8.3)	
	I use it really often	14 (23.3)	7 (58.3)	
50–59 (*N* = 35)	I don’t use it at all	7 (24.1)	2 (33.3)	0.29
	I hardly use it	1 (3.4)	1 (16.7)	
	I sometimes use it	3 (10.3)	0	
	I often use it	9 (31.0)	0	
	I use it really often	9 (31.0)	3 (50.0)	
60–69 (*N* = 19)	I don’t use it at all	9 (56.3)	2 (66.7)	1.00
	I hardly use it	0	0	
	I sometimes use it	3 (18.8)	0	
	I often use it	2 (12.5)	0	
	I use it really often	2 (12.5)	1 (33.3)	
70 < (N = 5)	I don’t use it at all	5 (100.0)	0	1.00
	I hardly use it	0	0	
	I sometimes use it	0	0	
	I often use it	0	0	
	I use it really often	0	0	

*: *p* < 0.05, **: *p* < 0.001

## Discussion

To our knowledge, this is the first study that investigated the prevalence of PIU, and degree of co-morbidity between PIU and psychiatric symptoms among adult psychiatric patients.

We identified 58 problematic internet users who scored either high IAT (40 and more) or CIUS (21 and more) out of 231 patients (25.1%). Although their demographic background was different, Osaki et al. [47] reported only 4.0% of people scored 40 and more on the IAT in the Japanese general population (*N* = 4.153). Bakken et al. [10] in Norway found percentages for at-risk internet users (YDQ score 3–4) and internet addiction (YDQ score 5–8) of 5.2% (177/3.393) and 1.0% (35/3.393) respectively. Zadra et al. [11] reported that 685 out of 15.023 German general population (4.5%) showed 21 points and more on the CIUS. Thus, the prevalence of problematic internet users among the general population might be estimated to be 4.0 to 6.2%. Bakken et al. [10] reported that 13.0% of their respondents did not use the internet at all, which means 6.0% of their internet using population displayed at-risk internet users, and 1.2% internet addiction. Considering these results, the prevalence of PIU among psychiatric patients could be much higher compared to that of general population.

There may be several reasons explaining the high prevalence of PIU among psychiatric patients. First, our study found that problematic internet users scored higher on scales measuring sleep problems, depression, trait anxiety, ADHD, autism, OCD, SAD, and impulsivity. This is in line with previous research in non-psychiatric students and adolescents [2, 3, 12]. The severity of these psychiatric symptoms in our samples may very well be higher than that of previous studies’ subjects because all of our subjects were diagnosed with at least one of primary psychiatric disorders. Therefore, it is reasonable to assume that the more severe the psychiatric illness of the studied population, the higher the prevalence of PIU. Then, some measurements used in our study such as STAI, AQ, and BIS assess the inherent characteristics of each subject, and these measurements also showed a significant difference between the two groups. Due to its cross-sectional nature, this study is unable to identify any causal relationships between PIU and psychiatric disorders [3], but intrinsic characteristics that predispose towards anxiety, autism, and impulsiveness may be the same characteristics that cause people to develop PIU. Further study to reveal the causal relationship between psychiatric symptoms and PIU is needed. In contrast to previous research [2], we did not find PIU to be associated with alcohol abuse. This is likely due to the small number of patients with alcohol abuse attending our psychiatric outpatient clinic, as these patients are often referred to other hospitals where specialized treatment is available for alcohol addicts and abusers. Further studies that include a larger number of alcohol addiction patients are necessary.

The study also showed a strong inverse association of PIU and age, which might be caused by the high amount of smartphone use among the younger population. There is also a number of studies done in student and adolescent populations which also show an inverse association between PIU and age [12]. An investigation done by the Japanese Ministry of Internal Affairs and Communications among Japanese high schoolers showed figures of 4.6% of high school students scoring 70 or higher on the IAT, and 55.2% scoring between 40 and 70 (*N* = 14.071) [48]. The group closest in age to these studies was our group of respondents between the ages of 20 and 29, which showed a percentage of 46.0 of problematic internet users. Due to the strong apparent inverse correlation between age and PIU, it is very well possible that psychiatric patients of high school age would score higher than older psychiatric patients or healthy high school students. Further studies should clarify the prevalence of PIU among adolescent patients with psychiatric illnesses.

There was no significant difference in time spent for work-related activities on the internet between the two groups, while problematic internet users spent significantly longer private time compared to normal internet users. Regarding time spent for various private activities on the internet, amount spent for activities such as blogging, social networking, and watching movies would be associated with PIU. These findings offer an important suggestion in clinical settings, as it shows that psychiatric patients need not avoid using the internet for work. Furthermore, physicians could suggest their patients to restrict their internet use to specific activities in order to prevent PIU. Lastly, patients with PIU seem to use their smartphone a lot after they have gone to bed, or when they wake up during sleep. Physicians could help their patients by discouraging this bad sleep hygiene.

This investigation is limited by the relatively small sample size. Only patients who were examined at the University Hospital, Kyoto Prefectural University of Medicine’s Psychiatry outpatient clinic were recruited. Due to the small population size, we were unable to divide our population into groups with a specific psychiatric diagnosis and analyse correlations with scores on the questionnaires. Another limitation is the cross-sectional nature of the study. Because we did not measure our population repeatedly over time, there can be no conclusions drawn regarding causality. Furthermore, this study using an online questionnaire is likely to have greatly improved response rates (69.4%) among people with familiarity to the internet, but prevented us from collecting data on patients who were not used to answering questionnaires online and who did not have internet connection. In our study, we also didn’t employ more advanced statistical methods such as bootstrapping to address the deviations from normality in our data. Finally, the questionnaire relied on self-report. One of the strengths of this study is that many different scales were filled in by each respondent, providing a clear and broad overview of any overlapping or co-morbid illness in every patient.

Future research could aim at finding causal relationships between PIU and various co-morbidities. Future research could also investigate the effectiveness of specialized treatment for psychiatric patients with PIU, due to the high degree of co-morbidity.

## Conclusion

This study in adult psychiatric patients suggest that the prevalence of PIU is higher (25%) than that of general population (6%). In addition, PIU in psychiatric patients is associated with higher scores on questionnaires investigating sleep problems, depression, trait anxiety, ADHD, autism, OCD, SAD, and impulsivity. More research is needed to determine the causal relation between PIU and psychiatric symptoms.

## Appendix

### Internet use and background questions

**Q1. Please answer how much time you spend on average per day doing online activities for the purpose of work or school (mailing, looking up information etc. all added up).**

Answers:

None, <30 min, 30 min – 1 h, 1 h - 3 h, 3 h - 5 h, >5 h.

**Q2. Please answer how much time you spend each day on average on the private online activities below not for the purpose of work or school**

1. Communication (Mail, Line, Skype etc.)
2. Looking up information (Google etc.)
3. Reading or watching the news
4. Reading or posting on blogs, forums, etc.
5. Reading or posting on social networking sites (Facebook, Twitter etc.)
6. Watching or posting on video sites (Youtube etc.)
7. Visiting adult sites
8. Downloading digital contents (videos, music, games etc.)
9. Internet auctions, shopping
10. Stocks, financial transactions (effects etc.)
11. Online games, social games
12. Other activities
13. The total of all private activities

Answers:

None, <30 min, 30 min – 1 h, 1 h - 3 h, 3 h - 5 h, >5 h.

**Q3. How much do you use each of the devices below when you access the internet for private purposes?**

1. Desktop PC
2. Notebook/laptop
3. Tablet (iPad etc.)
4. Smartphone
5. PHS or mobile phones other than smartphones
6. Music player (iPod etc.) or gaming console
7. Other

Answers:

I don’t use it at all, I hardly use it, I sometimes use it, I often use it, I use it really often.

**Q4. After you’ve gone to bed but before you sleep, how much on average per day do you use the smartphone or tablet?**

Answers:

Not at all, less than 10 min, between 10 and 30 min, between 30 min and 1 h, between 1 and 2 h, over 2 h.

**Q5. When you wake up during your sleep, how much do you use your smartphone or tabled in bed?**

Answers:

I don’t use it at all, I hardly use it, I sometimes use it, I often use it, I use it really often.

**Q6. Please choose what best fits your age from the options below.**

Answers: 20s, 30s, 40s, 50s, 60s, 70 or over.

**Q7. Please choose your gender.**

Answers: Male, Female, other (transgender).

**Q8. Please choose what best fits your highest level of education from the options below.**

Answers:

Junior high school, high school dropout, high school, university dropout, junior college, university, graduate school dropout, graduate school.

**Q9. Please choose what best fits your marital status.**

Answers:

Unmarried, married or living together, widowed, divorced, living apart, other.

**Q10. Please choose what best fits your work or school.**

Answers:

Working (full time), working (contracted), working (part-time), self-employed, professional, housewife/−husband, student, prep−/vocational student, not working at the moment, on leave, other.

**Q11. Please choose which of the people below you live together with.**

Answers:

Father, mother, siblings, grandfather, grandmother, child, grandchild, partner, father-in-law, mother-in-law, brother/sister-in-law, grandfather-in-law, grandmother-in-law, relatives, friends, other, alone, alone in an institution

## Additional file

Additional file 1: Table S1. Answers for “After you’ve gone to bed but before you sleep, how much on average per day do you use the smartphone or tablet?”. **Table S2.** Answers for “When you wake up during your sleep, how much do you use your smartphone or tabled in bed?”. **Table S3.** Reported time spent doing several private activities on the internet in problematic internet users compared with normal internet users. **Table S4.** Use frequency of different mediums to access internet. (DOCX 33 kb)

## Abbreviations

ADHD: attention deficit hyperactivity disorder
APA: American Psychiatric Association
AQ: The Autism Spectrum Quotient
ASRS: The Adult ADHD Self-report Scale
AUDIT: The Alcohol Use Disorders Identification Test
BDI-II: The Beck Depression Inventory II
BIS: The Barratt Impulsive Scale
CGI-S: The Clinical Global Impression Severity
CIUS: The Compulsive Internet Use Scale
DSM: Diagnostic and Statistical Manual of Mental Disorders
IAT: Young’s Internet Addiction Test
ICD-10: The tenth revision of the International Classification of Diseases and Related Health Problems
LSAS: The Liebowitz Social Anxiety Scale
OCD: Obsessive-compulsive disorder
OCI: The Obsessive-Compulsive Inventory
PIU: Problematic internet use
SAD: Social anxiety disorder
STAI: The State-Trait Anxiety Inventory
YDQ: The Young Diagnostic Questionnaire

## References

[1] Young KS (1996). Psychology of computer use: XL. Addictive use of the internet: a case that breaks the stereotype. Psychol Rep.

[2] Ho RC, Zhang MW, Tsang TY, Toh AH, Pan F, Lu Y, Cheng C, Yip PS, Lam LT, Lai CM (2014). The association between internet addiction and psychiatric co-morbidity: a meta-analysis. BMC Psychiatry.

[3] Carli V, Durkee T, Wasserman D, Hadlaczky G, Despalins R, Kramarz E, Wasserman C, Sarchiapone M, Hoven CW, Brunner R (2013). The association between pathological internet use and comorbid psychopathology: a systematic review. Psychopathology.

[4] Shaffer HJ, Hall MN, Vander Bilt J (2000). “Computer addiction”: a critical consideration. Am J Orthop.

[5] Subramaniam M (2014). Re-thinking internet gaming: from recreation to addiction. Addiction.

[6] American Psychiatric A, American Psychiatric Association DSMTF. Diagnostic and statistical manual of mental disorders : DSM-5. 5th ed: American Psychiatric Pub; 2013.

[7] Cheng C, Li AY (2014). Internet addiction prevalence and quality of (real) life: a meta-analysis of 31 nations across seven world regions. Cyberpsychol Behav Soc Netw.

[8] Tsitsika A, Janikian M, Schoenmakers TM, Tzavela EC, Olafsson K, Wojcik S, Macarie GF, Tzavara C, Richardson C (2014). Internet addictive behavior in adolescence: a cross-sectional study in seven European countries. Cyberpsychol Behav Soc Netw.

[9] Mak KK, Lai CM, Watanabe H, Kim DI, Bahar N, Ramos M, Young KS, Ho RC, Aum NR, Cheng C (2014). Epidemiology of internet behaviors and addiction among adolescents in six Asian countries. Cyberpsychol Behav Soc Netw.

[10] Bakken IJ, Wenzel HG, Gotestam KG, Johansson A, Oren A (2009). Internet addiction among Norwegian adults: a stratified probability sample study. Scand J Psychol.

[11] Zadra S, Bischof G, Besser B, Bischof A, Meyer C, John U, Rumpf HJ (2016). The association between internet addiction and personality disorders in a general population-based sample. J Behav Addict.

[12] Ko CH, Yen JY, Yen CF, Chen CS, Chen CC (2012). The association between internet addiction and psychiatric disorder: a review of the literature. Eur Psychiatry.

[13] Wei HT, Chen MH, Huang PC, Bai YM (2012). The association between online gaming, social phobia, and depression: an internet survey. BMC Psychiatry.

[14] Goel D, Subramanyam A, Kamath R (2013). A study on the prevalence of internet addiction and its association with psychopathology in Indian adolescents. Indian J Psychiatry.

[15] Huang AC, Chen HE, Wang YC, Wang LM (2014). Internet abusers associate with a depressive state but not a depressive trait. Psychiatry Clin Neurosci.

[16] Park S, Hong KE, Park EJ, Ha KS, Yoo HJ (2013). The association between problematic internet use and depression, suicidal ideation and bipolar disorder symptoms in Korean adolescents. Aust N Z J Psychiatry.

[17] Lam LT, Peng ZW (2010). Effect of pathological use of the internet on adolescent mental health: a prospective study. Arch Pediatr Adolesc Med.

[18] Dong G, Lu Q, Zhou H, Zhao X (2011). Precursor or sequela: pathological disorders in people with internet addiction disorder. PLoS One.

[19] Gentile DA, Choo H, Liau A, Sim T, Li D, Fung D, Khoo A (2011). Pathological video game use among youths: a two-year longitudinal study. Pediatrics.

[20] Van den Bulck J (2004). Television viewing, computer game playing, and internet use and self-reported time to bed and time out of bed in secondary-school children. Sleep.

[21] Dworak M, Schierl T, Bruns T, Struder HK (2007). Impact of singular excessive computer game and television exposure on sleep patterns and memory performance of school-aged children. Pediatrics.

[22] Shochat T, Flint-Bretler O, Tzischinsky O (2010). Sleep patterns, electronic media exposure and daytime sleep-related behaviours among Israeli adolescents. Acta Paediatr.

[23] Brunborg GS, Mentzoni RA, Molde H, Myrseth H, Skouveroe KJ, Bjorvatn B, Pallesen S (2011). The relationship between media use in the bedroom, sleep habits and symptoms of insomnia. J Sleep Res.

[24] World Health Organization (1992). The ICD-10 classification of mental and behavioural disorders : clinical descriptions and diagnostic guidelines.

[25] Young KS. Caught in the net : how to recognize the signs of internet addiction, and a winning strategy for recovery: Wiley; 1998.

[26] Meerkerk GJ, Van Den Eijnden RJ, Vermulst AA, Garretsen HF (2009). The compulsive internet use scale (CIUS): some psychometric properties. Cyberpsychol Behav.

[27] KFR Y (2012). The reliability and validity of three internet addiction instruments in the Japanese population.

[28] Guertler D, Rumpf HJ, Bischof A, Kastirke N, Petersen KU, John U, Meyer C (2014). Assessment of problematic internet use by the compulsive internet use scale and the internet addiction test: a sample of problematic and pathological gamblers. Eur Addict Res.

[29] Okajima I, Nakajima S, Kobayashi M, Inoue Y (2013). Development and validation of the Japanese version of the Athens insomnia scale. Psychiatry Clin Neurosci.

[30] Soldatos CR, Dikeos DG, Paparrigopoulos TJ (2000). Athens insomnia scale: validation of an instrument based on ICD-10 criteria. J Psychosom Res.

[31] Beck AT, Steer RA, Brown G (1996). Manual for the Beck depression inventory-II.

[32] Kojima M, Furukawa TA, Takahashi H, Kawai M, Nagaya T, Tokudome S (2002). Cross-cultural validation of the Beck depression inventory-II in Japan. Psychiatry Res.

[33] Spielberger CD, Reheiser EC (2009). Assessment of emotions: anxiety, anger, depression, and curiosity. Applied Psychology: Health and Well-Being.

[34] Nakazato K, Mizuguchi T (1982). Development and validation of Japanese version of state-trait anxiety inventory: a study with female subjects. hinshin Igaku [in Japanese].

[35] Kessler RC, Adler LA, Gruber MJ, Sarawate CA, Spencer T, Van Brunt DL (2007). Validity of the World Health Organization adult ADHD self-report scale (ASRS) screener in a representative sample of health plan members. Int J Methods Psychiatr Res.

[36] Woodbury-Smith MR, Robinson J, Wheelwright S, Baron-Cohen S (2005). Screening adults for Asperger syndrome using the AQ: a preliminary study of its diagnostic validity in clinical practice. J Autism Dev Disord.

[37] Wakabayashi A, Tojo Y, Baron-Cohen S, Wheelwright S (2004). The autism-Spectrum quotient (AQ) Japanese version: evidence from high-functioning clinical group and normal adults. Shinrigaku Kenkyu.

[38] Foa EB, Kozak MJ, Salkovskis PM, Coles ME, Amir N (1998). The validation of a new obsessive-compulsive disorder scale: the obsessive-compulsive inventory. Psychol Assess.

[39] Ishikawa R, Kobori O, Shimizu E (2014). Development and validation of the Japanese version of the obsessive-compulsive inventory. BMC Res Notes.

[40] Heimberg RG, Horner KJ, Juster HR, Safren SA, Brown EJ, Schneier FR, Liebowitz MR (1999). Psychometric properties of the Liebowitz social anxiety scale. Psychol Med.

[41] Asakura S, Inoue S, Sasaki F, Sasaki Y, Kitagawa N, Inoue T, Denda K, Koyama T (2002). Reliability and validity of the Japanese version of the Liebowitz social anxiety scale. Seishin Igaku [in Japanese].

[42] Daeppen JB, Yersin B, Landry U, Pecoud A, Decrey H (2000). Reliability and validity of the alcohol use disorders identification test (AUDIT) imbedded within a general health risk screening questionnaire: results of a survey in 332 primary care patients. Alcohol Clin Exp Res.

[43] Kawada T, Inagaki H, Kuratomi Y (2011). The alcohol use disorders identification test: reliability study of the Japanese version. Alcohol.

[44] Patton JH, Stanford MS, Barratt ES (1995). Factor structure of the Barratt impulsiveness scale. J Clin Psychol.

[45] Someya T, Sakado K, Seki T, Kojima M, Reist C, Tang SW, Takahashi S (2001). The Japanese version of the Barratt impulsiveness scale, 11th version (BIS-11): its reliability and validity. Psychiatry Clin Neurosci.

[46] Busner J, Targum SD (2007). The clinical global impressions scale: applying a research tool in clinical practice. Psychiatry (Edgmont).

[47] Osaki Y, Kinjo A (2015). Epidemiology on addictive disorders and behaviors in Japan. Nippon Rinsho [in Japanese].

[48] Ministry of Internal Affairs and Communications (2014). The survey report of smartphone-app usage and tendency of internet addiction among Japanese high school students. [in Japanese].

