# Supplementary material for: Problematic internet use and psychiatric co-morbidity in a population of Japanese adult psychiatric patients
Source: BMC Psychiatry. 2018 Jan 17;18:9. doi: 10.1186/s12888-018-1588-z (PMC5773124; doi:10.1186/s12888-018-1588-z)
Supplement: Additional file 1: Table S1. — Answers for “After you’ve gone to bed but before you sleep, how much on average per day do you use the smartphone or tablet?”. Table S2. Answers for “When you wake up during your sleep, how much do you use your smartphone or tabled in bed?”. Table S3. Reported time spent doing several private activities on the internet in problematic internet users compared with normal internet users. Table S4. Use frequency of different mediums to access internet. (DOCX 33 kb) [file 12888_2018_1588_MOESM1_ESM.docx]

Table S1. Answers for “After you’ve gone to bed but before you sleep, how much on average per day do you use the smartphone or tablet?”.

| **Normal internet users, n (%) (N=173)** | | | | | |  | **Problematic internet users, n (%) (N=58)** | | | | | |  | ***p* (Mann-Whitney U test)** |
| --- | --- | --- | --- | --- | --- | --- | --- | --- | --- | --- | --- | --- | --- | --- |
| None | <10m | 10m-30m | 30m-1h | 1h-2h | >2h |  | None | <10m | 10m-30m | 30m-1h | 1h-2h | >2h |  |  |
| 68  (39.3) | 35  (20.2) | 30  (17.3) | 28  (16.2) | 8  (4.6) | 4  (2.3) |  | 16  (27.6) | 5  (8.6) | 7  (12.1) | 10  (17.2) | 9  (15.5) | 11  (19.0) |  | **<0.001** |

Table S2. Answers for “When you wake up during your sleep, how much do you use your smartphone or tabled in bed?”.

| **Use frequency** | **Normal internet users, n (%) (N=173)** | **Problematic internet users, n (%) (N=58)** | ***p* (Fisher’s exact test)** |
| --- | --- | --- | --- |
| I don’t use it at all | 99 (57.2) | 21 (36.2) | **<0.001** |
| I hardly use it | 35 (20.2) | 9 (15.5) |  |
| I sometimes use it | 28 (16.2) | 13 (22.4) |  |
| I often use it | 8 (4.6) | 5 (8.6) |  |
| I use it really often | 3 (1.7) | 10 (17.2) |  |

Table S3. Reported time spent doing several private activities on the internet in problematic internet users compared with normal internet users.

| **Private Activities** |  | **Normal internet users, n (%) (N=173)** | | | | | |  | **Problematic internet users, n (%) (N=58)** | | | | | |  | ***p* (Mann-Whitney U test)** |
| --- | --- | --- | --- | --- | --- | --- | --- | --- | --- | --- | --- | --- | --- | --- | --- | --- |
|  |  | None | <30m | 30m-1h | 1h-3h | 3h-5h | >5h |  | None | <30m | 30m-1h | 1h-3h | 3h-5h | >5h |  |  |
| Communication |  | 28  (16.2) | 98  (53.8) | 32  (18.5) | 14  (8.1) | 5  (2.9) | 1  (0.6) |  | 7  (12.1) | 26  (44.8) | 11  (19.0) | 8  (13.8) | 1  (1.7) | 5  (8.6) |  | **0.040** |
| Information |  | 11  (6.4) | 86  (49.7) | 47  (27.2) | 25  (14.5) | 2  (1.2) | 2  (1.2) |  | 2  (3.4) | 19  (32.8) | 19  (32.8) | 12  (20.7) | 3  (5.2) | 3  (5.2) |  | **0.003*** |
| News |  | 30  (17.3) | 91  (52.6) | 40  (23.1) | 9  (5.2) | 1  (0.6) | 2  (1.2) |  | 6  (10.3) | 33  (56.9) | 12  (20.7) | 4  (6.9) | 0  (0.0) | 3  (5.2) |  | 0.279 |
| Blogging |  | 88  (50.9) | 56  (32.4) | 18  (10.4) | 9  (5.2) | 1  (0.6) | 1  (0.6) |  | 10  (17.2) | 21  (36.2) | 12  (20.7) | 9  (15.5) | 2  (3.4) | 4  (6.9) |  | **<0.001*** |
| Social networks |  | 89  (51.4) | 61  (35.3) | 12  (6.9) | 10  (5.8) | 1  (0.6) | 0  (0.0) |  | 19  (32.8) | 14  (24.1) | 8  (13.8) | 12  (20.7) | 5  (8.6) | 0  (0.0) |  | **<0.001*** |
| Movie/Music |  | 65  (37.6) | 71  (41.0) | 19  (11.0) | 13  (7.5) | 4  (2.3) | 1  (0.6) |  | 13  (22.4) | 16  (27.6) | 11  (19.0) | 10  (17.2) | 4  (6.9) | 4  (6.9) |  | **<0.001*** |
| Pornography |  | 148  (85.5) | 24  (13.9) | 1  (0.6) | 0  (0.0) | 0  (0.0) | 0  (0.0) |  | 43  (74.1) | 8  (13.8) | 4  (6.9) | 2  (3.4) | 1  (1.7) | 0  (0.0) |  | **0.024** |
| Downloading |  | 114  (65.9) | 46  (26.6) | 8  (4.6) | 5  (2.9) | 0  (0.0) | 0  (0.0) |  | 35  (60.3) | 15  (25.9) | 3  (5.2) | 4  (6.9) | 0  (0.0) | 1  (1.7) |  | 0.307 |
| Auctions |  | 81  (46.8) | 68  (39.3) | 16  (9.2) | 7  (4.0) | 1  (0.6) | 0  (0.0) |  | 20  (34.5) | 24  (41.4) | 5  (8.6) | 7  (12.1) | 1  (1.7) | 1  (1.7) |  | **0.034** |
| Stocks |  | 155  (89.6) | 13  (7.5) | 3  (1.7) | 1  (0.6) | 0  (0.0) | 1  (0.6) |  | 54  (93.1) | 1  (1.7) | 0  (0.0) | 1  (1.7) | 0  (0.0) | 2  (3.4) |  | 0.492 |
| Games |  | 119  (68.8) | 25  (14.5) | 15  (8.7) | 13  (7.5) | 1  (0.6) | 0  (0.0) |  | 32  (55.2) | 5  (8.6) | 6  (10.3) | 8  (13.8) | 4  (6.9) | 3  (5.2) |  | **0.008** |
| Other |  | 137  (79.2) | 29  (16.8) | 5  (2.9) | 2  (1.2) | 0  (0.0) | 0  (0.0) |  | 38  (65.5) | 12  (20.7) | 3  (5.2) | 3  (5.2) | 0  (0.0) | 2  (3.4) |  | **0.019** |

* Significant after Bonferroni correction

Table S4. Use frequency of different mediums to access internet

| **Medium** | **Use frequency** | **Normal internet users, n (%) (N=173)** | **Problematic internet users, n (%) (N=58)** | ***p* (Fisher’s exact test)** |
| --- | --- | --- | --- | --- |
| Desktop | I don’t use it at all | 117 (67.6) | 32 (55.2) | 0.39 |
|  | I hardly use it | 16 (9.2) | 8 (13.8) |  |
|  | I sometimes use it | 14 (8.1) | 8 (13.8) |  |
|  | I often use it | 10 (5.8) | 3 (5.2) |  |
|  | I use it really often | 16 (9.2) | 7 (12.1) |  |
| Laptop | I don’t use it at all | 63 (36.4) | 25 (43.1) | 0.12 |
|  | I hardly use it | 24 (13.9) | 8 (13.8) |  |
|  | I sometimes use it | 34 (19.7) | 5 (8.6) |  |
|  | I often use it | 33 (19.1) | 8 (13.8) |  |
|  | I use it really often | 19 (11.0) | 12 (20.7) |  |
| Tablet | I don’t use it at all | 111 (64.2) | 37 (63.8) | 0.36 |
|  | I hardly use it | 9 (5.2) | 5 (8.6) |  |
|  | I sometimes use it | 24 (13.9) | 6 (10.3) |  |
|  | I often use it | 15 (8.7) | 2 (3.4) |  |
|  | I use it really often | 14 (8.1) | 8 (13.8) |  |
| Smartphone | I don’t use it at all | 48 (27.7) | 13 (22.4) | **<0.001*** |
|  | I hardly use it | 8 (4.6) | 1 (1.7) |  |
|  | I sometimes use it | 21 (12.1) | 0 (0.0) |  |
|  | I often use it | 40 (23.1) | 2 (3.4) |  |
|  | I use it really often | 56 (32.4) | 42 (72.4) |  |
| Mobile phone | I don’t use it at all | 130 (75.1) | 49 (84.5) | 0.28 |
|  | I hardly use it | 13 (7.5) | 6 (10.3) |  |
|  | I sometimes use it | 14 (8.1) | 1 (1.7) |  |
|  | I often use it | 8 (4.6) | 1 (1.7) |  |
|  | I use it really often | 8 (4.6) | 1 (1.7) |  |
| Music player | I don’t use it at all | 126 (72.8) | 41 (70.7) | 0.10 |
| /Gaming console | I hardly use it | 14 (8.1) | 7 (12.1) |  |
|  | I sometimes use it | 24 (13.9) | 4 (6.9) |  |
|  | I often use it | 7 (4.0) | 2 (3.4) |  |
|  | I use it really often | 2 (1.2) | 4 (6.9) |  |
| Others | I don’t use it at all | 161 (93.1) | 53 (91.4) | 0.80 |
|  | I hardly use it | 8 (4.6) | 4 (6.9) |  |
|  | I sometimes use it | 4 (2.3) | 1 (1.7) |  |
|  | I often use it | 0 | 0 |  |
|  | I use it really often | 0 | 0 |  |

* Significant after Bonferroni correction
